# Membrane Permeabilization by Oligomeric α-Synuclein: In Search of the Mechanism

**DOI:** 10.1371/journal.pone.0014292

**Published:** 2010-12-13

**Authors:** Bart D. van Rooijen, Mireille M. A. E. Claessens, Vinod Subramaniam

**Affiliations:** 1 Nanobiophysics, MESA+ Institute for Nanotechnology, University of Twente, Enschede, Netherlands; 2 MIRA Institute for Biomedical Technology and Technical Medicine, University of Twente, Enschede, Netherlands; Uppsala University, Sweden

## Abstract

**Background:**

The question of how the aggregation of the neuronal protein α-synuclein contributes to neuronal toxicity in Parkinson's disease has been the subject of intensive research over the past decade. Recently, attention has shifted from the amyloid fibrils to soluble oligomeric intermediates in the α-synuclein aggregation process. These oligomers are hypothesized to be cytotoxic and to permeabilize cellular membranes, possibly by forming pore-like complexes in the bilayer. Although the subject of α-synuclein oligomer-membrane interactions has attracted much attention, there is only limited evidence that supports the pore formation by α-synuclein oligomers. In addition the existing data are contradictory.

**Methodology/Principal Findings:**

Here we have studied the mechanism of lipid bilayer disruption by a well-characterized α-synuclein oligomer species in detail using a number of *in vitro* bilayer systems and assays. Dye efflux from vesicles induced by oligomeric α-synuclein was found to be a fast all-or-none process. Individual vesicles swiftly lose their contents but overall vesicle morphology remains unaltered. A newly developed assay based on a dextran-coupled dye showed that non-equilibrium processes dominate the disruption of the vesicles. The membrane is highly permeable to solute influx directly after oligomer addition, after which membrane integrity is partly restored. The permeabilization of the membrane is possibly related to the intrinsic instability of the bilayer. Vesicles composed of negatively charged lipids, which are generally used for measuring α-synuclein-lipid interactions, were unstable to protein adsorption in general.

**Conclusions/Significance:**

The dye efflux from negatively charged vesicles upon addition of α-synuclein has been hypothesized to occur through the formation of oligomeric membrane pores. However, our results show that the dye efflux characteristics are consistent with bilayer defects caused by membrane instability. These data shed new insights into potential mechanisms of toxicity of oligomeric α-synuclein species.

## Introduction

The aggregation of the neuronal protein α-synuclein (αS) is implicated to be involved in the pathogenesis of Parkinson's disease. Aggregated insoluble αS is the main component of Lewy bodies, intracellular inclusion bodies which are the pathological characteristic of Parkinson's disease [1]. Furthermore, mutations and multiplications in the gene encoding αS have been identified to lead to familial forms of Parkinson's disease [2], [3], [4], [5], [6]. How the aggregation of αS is related to neuronal degeneration is an important unresolved question. In recent years attention has shifted to the early stages of the aggregation process of αS [7]. Oligomeric intermediates in the aggregation of αS have been found to be more toxic to cells than monomeric or fibrillar forms of the protein [8], [9]. A possible mechanism by which oligomers could be toxic is through the disruption and permeabilization of cellular membranes [10], [11], [12]. Oligomeric αS has been shown to permeabilize negatively charged synthetic phospholipid vesicles [12], [13]. We have recently studied similar oligomeric species that bind and permeabilize negatively charged vesicles [14], [15]. The solute efflux from these vesicles has been found to be selective for the molecular weight of the entrapped marker and might thus occur through a pore-like mechanism [16]. Formation of pore-like complexes by αS is supported by the observation of donut shaped protein particles by electron microscopy and AFM [17], [18]. However, there are many ways in which proteins can destabilize the membrane integrity [19] and the mechanisms by which oligomers cause membrane permeabilization have not been unambiguously determined. For instance, a thorough electrophysiological characterization of the hypothesized αS pore is still lacking.

In this article we present a range of biophysical experiments aimed at elucidating the mechanism by which oligomeric αS induces membrane permeabilization. Results from dynamic light scattering (DLS), confocal microscopy and a fluorescence re-quenching assay indicate that oligomer-induced permeabilization of vesicles occurs in a rapid all-or-none fashion in which the overall vesicle morphology is not changed. However, although vesicles appear intact, the vesicle disruption may be caused by the intrinsic instability of the highly charged membranes; negatively charged vesicles proved unstable to the adsorption of different unrelated proteins. A newly developed method based on a dextran coupled dye and an external quencher allows us to measure the permeability of the membrane as a function of equilibration time after the addition of oligomeric αS. The high molecular weight dextran molecules could escape the vesicle interior, pointing to the presence of large bilayer defects. In the presence of calcium, the oligomer induced vesicle disruption became sensitive to the molecular weight of the entrapped marker. However, even for the calcium-stabilized vesicles, transient membrane defects appeared to be the primary means of vesicular content loss. In summary, our results indicate that non-equilibrium phenomena contribute to the bilayer disruption process, which indicates that defects, rather than well-defined oligomer pores, likely dominate the observed membrane permeabilization.

## Results

It has been suggested that membrane permeabilization by oligomeric αS occurs through the insertion of pore-like complexes in the membrane [16]. The planar lipid bilayer technique is well suited to characterize proteins that form ion permeable pores in lipids [20] and could provide unambiguous evidence for the proposed pore model. However, the technique is extremely sensitive and prone to artifacts, as the observed activities are essentially single molecule events. To prove a pore-like model, a thorough, reproducible study is necessary.

**Figure 1:**
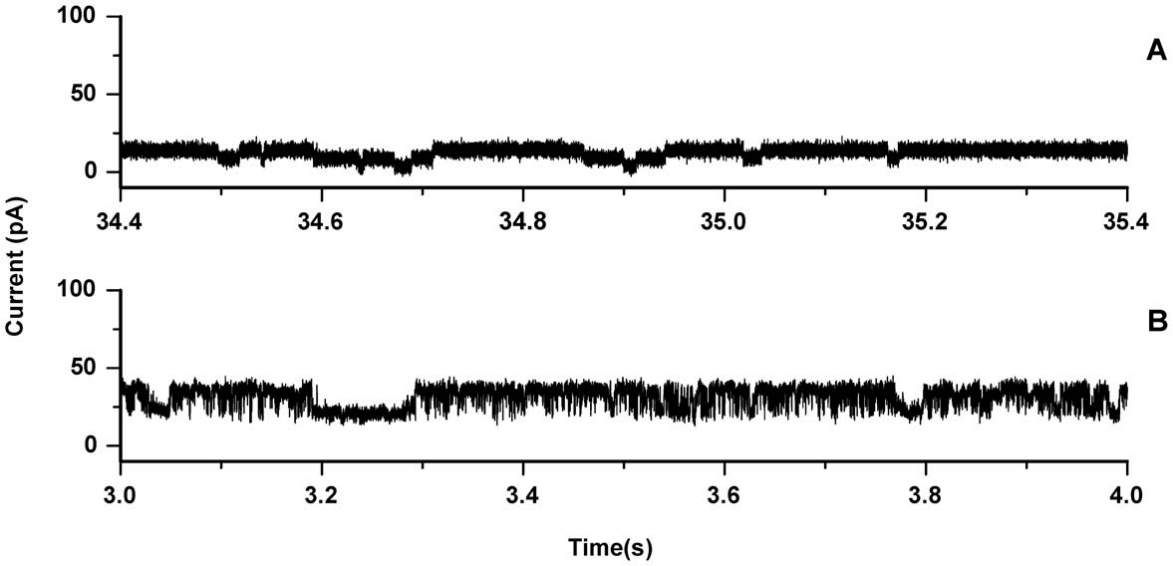
Recordings of channel events in a POPG planar lipid bilayer. The current was recorded with a transmembrane potential of 100 mV in the presence of 500 nM oligomeric αS. Current jumps were only observed if the membrane was formed and broken repeatedly. The top and bottom panels show the events observed in two separate experiments.

We and others have demonstrated that large unilamellar vesicles (LUVs) of 1-palmitoyl,2-oleoyl phosphatidylglycerol (POPG) are vulnerable to permeabilization by oligomeric αS [15], [16]. Therefore, POPG was used to create planar lipid bilayers. The monolayer folding technique was used to create solvent free lipid bilayers [20]. The resulting POPG bilayers were only stable for a short time, generally a few minutes to less than a minute (at a membrane voltage of 100 mV), thereby making it very difficult to assess the effect of the protein on membrane stability. After a stable bilayer was obtained a small amount of oligomeric protein (final concentration 200 nM) was added to the solution on both the cis- and trans-side of the membrane and the solution was thoroughly mixed. No current jumps characteristic of insertion of pore-like proteins were observed under these conditions. However if the membrane was broken and reformed repeatedly by lowering and raising the water-air interface, while mixing intermittently, sometimes events that could represent pore insertion were observed (Figure 1). However, these events were very difficult to reproduce, partly because the conditions required for these events also induce membrane instability. The insertion events were analyzed in more depth using the QuB software suite [21]. Comparing the results between separate experiments did not reveal a single conductance level or characteristic kinetics. For instance the events shown in Figure 1A showed a conductance level of around 0.05 nS with a long average lifetime of around 129 ms. The experiment shown in Figure 1B revealed conductance levels of 0.14 nS and 0.08 nS with fast kinetics with a lifetime of around 1.0 ms. Given the susceptibility to artifacts of this method, good statistics and reproducibility are necessary for reliable conclusions. Therefore, these results are not sufficient to prove a pore-like mechanism of disruption. Moreover, we have observed similar current jumps in POPG bilayers in the absence of protein, which were attributed to subtle hydrostatic pressure differences between the cis- and trans-chambers.

**Figure 2:**
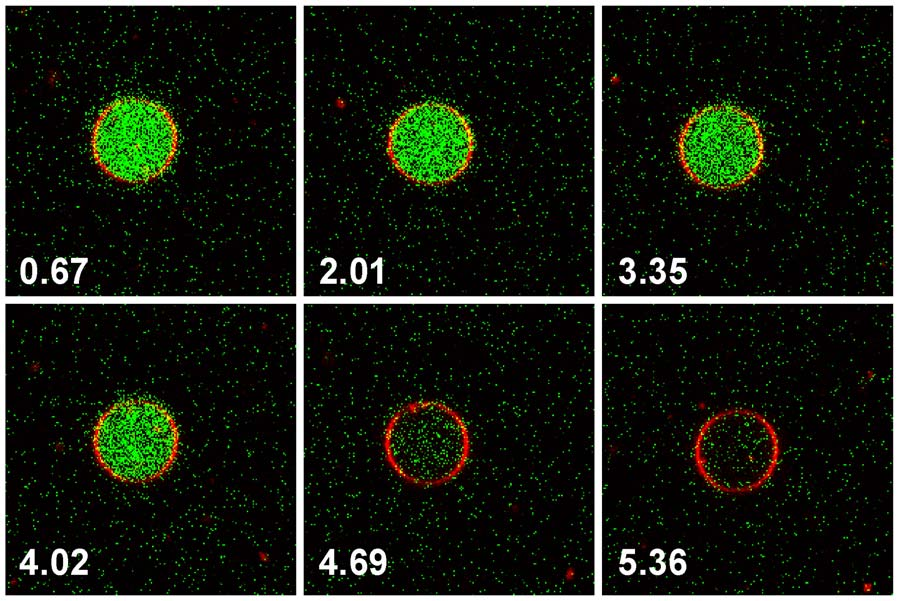
Confocal microscopy images of POPG GUVs filled with the dye HPTS (green). The quencher DPX was present on the outside of the vesicles. The GUV membrane was stained with DOPE-Rhodamine (red). The consecutive images (time stamp in seconds) show the kinetics of dye efflux from a single GUV. These data are also presented as a movie (see Movie S1).

To gain more insight into the mechanism of the disruption process, confocal fluorescence microscopy was used to observe the oligomer induced membrane permeability of giant unilamellar vesicles (GUVs). POPG GUVs encapsulating the dye 8-hydroxypyrene-1,3,6-trisulfonic acid (HPTS) were prepared and Rhodamine labeled lipid was added to visualize the lipid bilayer. The GUVs were diluted into salt containing buffer solutions. To further reduce the signal from un-encapsulated HPTS, the quencher p-Xylene-bis, N-pyridinium bromide (DPX) was added to the solution outside the GUVs. The resulting GUV solutions were stable and showed good encapsulation of HPTS. When oligomeric αS was added to the imaging chamber, the fluorescence from the vesicle interior was lost, either through HPTS efflux or DPX influx. By continuously imaging the GUVs just after oligomer addition, the leakage process could be followed in time (a movie is available online). Figure 2 shows several consecutive images after the addition of oligomeric αS. The images show that the leakage process is fast and that vesicles appear morphologically unchanged. These data are also presented as a movie (see Movie S1).

**Figure 3:**
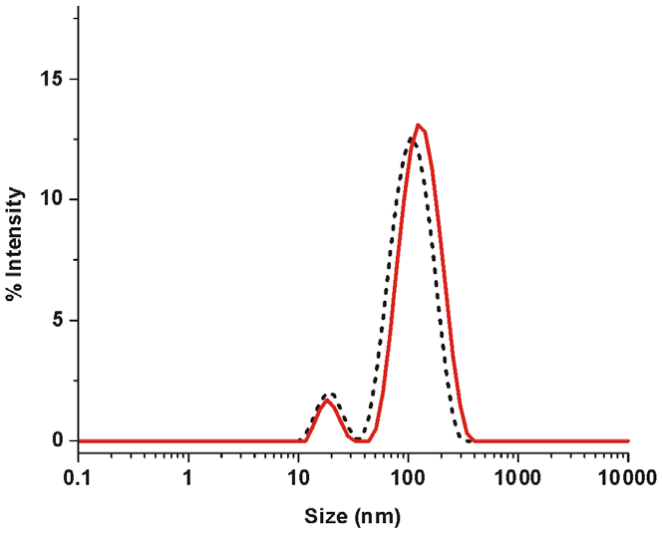
DLS on POPG LUVs at a lipid concentration of 20 µM in the presence of 1 µM oligomeric αS. Size distributions were acquired at different time points after oligomer addition: t = 0 (black dotted line), t = 15 minutes (red solid line).

To confirm that vesicles remain largely intact upon oligomer addition dynamic light scattering (DLS) experiments were performed. αS oligomers were added to POPG LUVs and the particle size distribution was followed over time. Figure 3 shows the characteristic size distribution of LUVs in the presence of oligomeric αS. Just after the addition of oligomers (t = 0), two distinct peaks corresponding to a size of around 18 nm for oligomeric αS and 110 nm for the POPG LUVs were observed. After 30 minutes both peaks were still present in the size distribution. The vesicle peak was shifted to slightly larger sizes of around 120 nm. The amount of light scattering from particles increases with increasing size of the particles. Corresponding to the shift in the particle size distribution, the photon count rate of the scattered light increased during the incubation time. The increase in size could be caused by an adsorbed protein layer on the outside of the vesicles, or an increase in surface area of the membrane surface due to protein insertion. Clearly the LUVs are not completely destroyed or solubilized by oligomeric αS, but remain largely intact over the course of the experiment.

**Figure 4:**
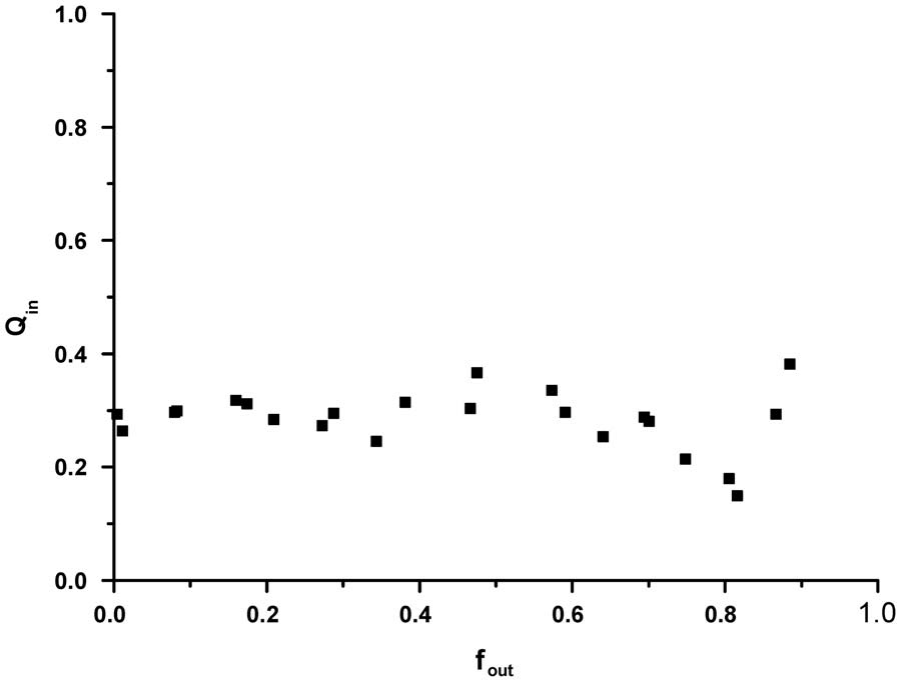
Fluorescence quenching assay results. POPG LUVs encapsulating HPTS/DPX were mixed with different amounts of oligomeric αS to a final lipid concentration of 25 µM. The quenching ratio *Q_in_* in the vesicle interior does not change as a function of the amount of escaped dye *f_out_*, which indicates that dye efflux from the LUVs occurs through an all-or-none mechanism.

The mechanism by which LUVs release their content upon interaction with oligomeric αS was further studied by using a re-quenching assay based on the HPTS/DPX dye quencher pair. Dye release can occur either though an all-or-none or a graded mechanism [22]. In other words: if half of the encapsulated solute has escaped, this can either be the result of half of the vesicles releasing all their content, or all vesicles releasing half of their content. The dye quencher pair HPTS/DPX was co-encapsulated inside LUVs, which causes the fluorescence of HPTS to be quenched by a certain ratio Q_in_. In the case of all-or-none release, the ratio Q_in_ is independent of the fraction of escaped solute f_out_. In the case of graded release, assuming there is no preferential release of HPTS over DPX, the ratio Q_in_ depends on the fraction of escaped solute, since this causes the DPX concentration to drop. The quenching ratio inside the vesicles as a function of the amount of escaped solute is experimentally accessible and was obtained by titrating DPX to LUVs after solute efflux has occurred, as is described in the Materials and Methods section. In the experiment, POPG LUVs were incubated with different concentrations of oligomeric αS, after which Q_in_ and f_out_ were subsequently determined. The results in Figure 4 show that Q_in_ remains roughly constant with the increase of the escaped fraction of dye f_out_, indicating that leakage occurs through an all-or-none mechanism. This result supports the data from the confocal microscopy experiment, in which dye efflux from GUVs was extremely rapid, and proceeded until the vesicle fluorescence intensity inside and outside the GUV were similar.

**Figure 5:**
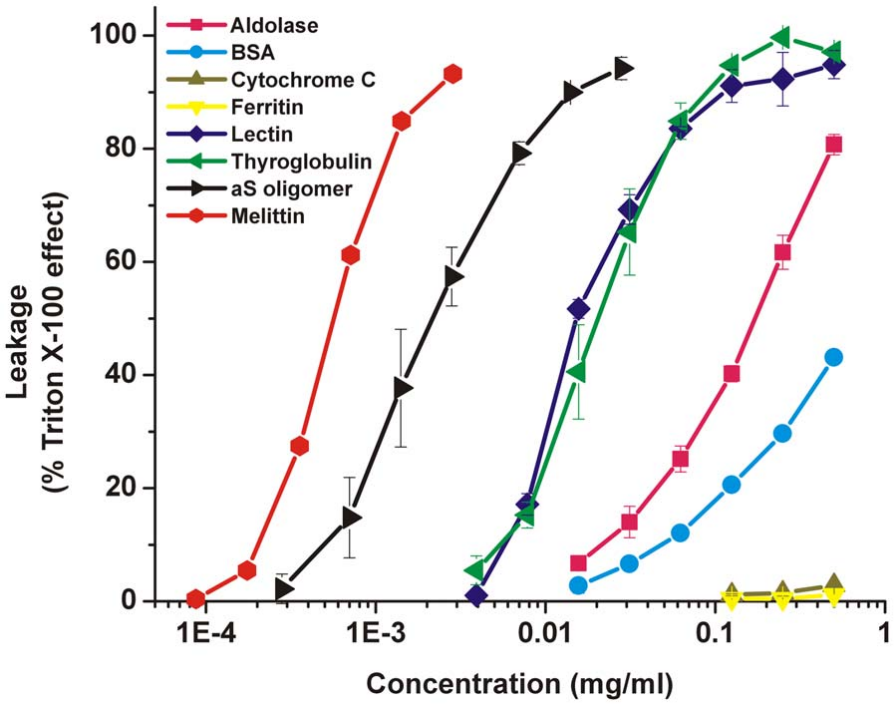
Stability of POPG LUVs. Stability of POPG LUVs to protein absorption was measured by the calcein efflux induced by the addition of different concentrations of several proteins at a phospholipid concentration of 20 µM. The error bars indicate the standard deviation (n = 3).

Since calcein efflux has also been observed upon addition of monomeric and fibrillar αS [14], disruption of the vesicles might represent a measure of intrinsic vesicle instability to protein adsorption rather than a specific mechanism. Therefore, the stability of POPG LUVs to protein adsorption was investigated by adding a number of unrelated proteins. Proteins were selected to have a range of different properties such as molecular mass, charge and biological function. The results are summarized in Figure 5. Bovine cytochrome c (MW 12.3 kDa, pI 9.3) and horse apoferritin (MW 443 kDa, pI 4.3) did not significantly affect vesicle stability. Rabbit aldolase (168 kDa, pI 8.1) and bovine serum albumin (66 kDa, pI 4.9) were able to disrupt 50% of the POPG LUVs at high concentrations of around 0.2 and 0.5 mg/ml respectively. Bovine thyroglobulin (660 kDa, pI 4.5) and lectin from *Lens culinaris* (24 kDa, pI 8.5) were even more effective in inducing calcein efflux from the LUVs with 50% efflux at approximately 0.02 mg/ml. As a positive control, the membrane disrupting peptide melittin from honey bee venom, induced a 50% leakage at around 0.0005 mg/ml. Oligomeric αS induced 50% leakage at ∼0.0018 mg/ml. No correlation of the induced leakage to the protein size or charge was observed. Thus specific structural features rather than broad biochemical characteristics determine the disruptive effect. Oligomeric αS was more effective in disrupting POPG LUVs than many of the other proteins used, and was around 4 times less effective than melittin, which is a well-characterized membrane disrupting peptide. Although disruption of LUVs is thus most likely related to the structural properties of the αS oligomer, vesicle instability to protein absorption clearly is an issue for negatively charged LUVs.

**Figure 6:**
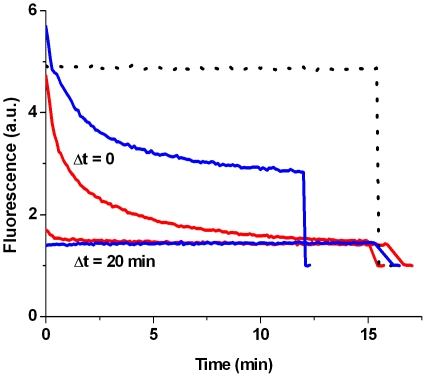
Transient vesicle permeabilization assay. To measure if vesicle permeabilization was transient POPG LUVs encapsulating dextran-PTS were prepared. The quencher DPX was added Δt after mixing the vesicles with oligomeric αS to a final concentration of 0.25 mM lipid and 1.5 µM αS. The dotted line is a background curve in the absence of protein. After ∼15 minutes Triton X-100 is added to disrupt all vesicles, the curves are normalized to this final value. In the absence of calcium the dextran-PTS conjugate escapes the vesicle interior upon protein interaction (3kDa red lines, 70 kDa blue lines).

The results that negatively charged LUVs are unstable to protein adsorption, that vesicle efflux from a single GUV is rapid, and that leakage occurs through an all-or-none mechanism, led us to hypothesize that the dye efflux occurs as a result of a transient non-equilibrium bilayer disruption process. Membrane defects could appear upon binding of the oligomers to the membrane. The bilayer might be perturbed by the increased surface area in the outer leaflet upon protein adsorption. After a certain period the bilayer will have accommodated the protein and may reach a new equilibrium state in which no defects are present. To be able to assess the permeability of the membrane at a certain time point after the addition of oligomeric αS, a new assay was developed. An isothiocyanate derivative of HPTS was conjugated to amine-modified dextran of different molecular weights (3kDa, 10 kDa and 70 kDa). If the membrane defects are small, the dextran-PTS conjugate will stay encapsulated in the LUV interior. Membrane permeability can subsequently be measured by adding DPX, which quenches the dextran-PTS fluorescence if it can cross the vesicle membrane. In the experiment POPG LUVs containing the dextran-PTS conjugate were mixed with oligomeric αS. DPX was either present from the start of the experiment or added after a certain reaction time. Figure 6 shows how the dextran-PTS fluorescence changes over time. If DPX is present from the start of the experiments the fluorescence is slowly quenched and equilibrium is only reached after 10–15 minutes. However if the LUVs were preincubated with oligomeric αS for 20 minutes the fluorescence was immediately lost on DPX addition. This indicates that the dextran conjugate most likely has escaped from the vesicle interior. This was also the case for the 70 kDa dextran conjugate indicating that the initial membrane defects are large.

**Figure 7:**
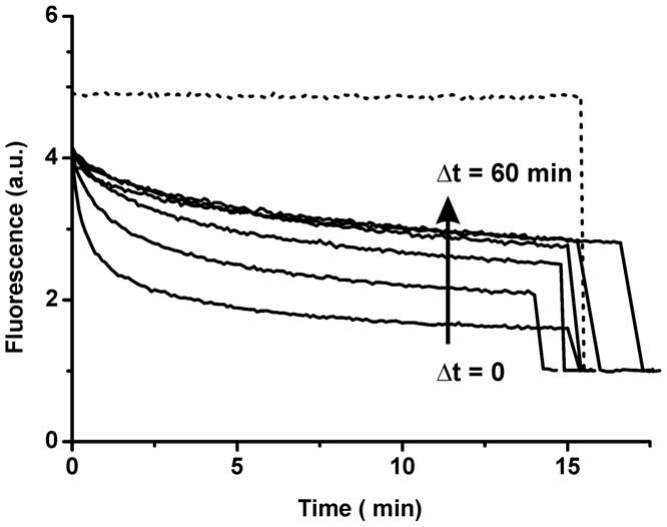
POPG LUVs encapsulating a 3 kDa dextran-PTS. In the presence of 5 mM Ca^2+^ the 3Kda dextran-PTS remains in the vesicle interior. The quencher DPX was added Δt after mixing the vesicles with oligomeric αS to a final concentration of 0.25 mM lipid and 1.5 µM αS. The amount of DPX influx is reduced upon increasing equilibration time (Δt = 0, 0.5, 1, 5, 20 and 60 minutes). The dotted line is a background curve in the absence of protein. After approximately 15 minutes Triton X-100 is added to disrupt all vesicles, and all curves are normalized to the value obtained after Triton disruption.

It has been reported that solute efflux from negatively charged vesicles induced by oligomeric αS is size selective in the presence of calcium ions [16]. Figure 7 shows the results for the time dependent membrane permeability to DPX in the presence of calcium. When oligomeric αS, LUVs and DPX were mixed at the same time, a clear decrease in fluorescence intensity was observed. If the LUVs were preincubated with oligomeric αS for a certain time before DPX addition, the majority of dextran-dye conjugate was retained in the vesicle interior. This allows us to map the permeability of the membrane to DPX as a function of incubation time of the LUV-oligomer mixture. The results indicate that the membrane permeability is largest immediately after addition of the αS oligomers. The initial defects seem to heal swiftly and permeability goes down within 5 minutes to a stable level. However, this level of permeability was not reduced any further even after 60 minutes of equilibration time of LUVs. This implies that in the presence of calcium the vesicle permeabilization is not a transient process. However, non-equilibrium phenomena in the first minute after oligomer addition to LUVs have a major contribution on the observed membrane permeability.

## Discussion

Oligomeric αS species have been prepared in a number of ways [8], [12], [23], [24]. However, little is known about the structural properties of these oligomers and the mechanism by which they are formed. In this study we have used a high concentration of monomeric αS to induce oligomerization and have isolated oligomeric species by size exclusion chromatography. This approach is relatively well characterized [14], [15], [25] and results in oligomers that have vesicle binding and disruption characteristics similar to those reported in the initial studies of oligomer induced membrane permeabilization [12], [16], [18]. It has been hypothesized that a possible mechanism by which these αS oligomers cause membrane permeabilization is the formation of pore-like structures in the membrane. However, this hypothesis is primarily supported by the result of a size selectivity of marker efflux in a vesicle permeabilization assay [16].

Single channel recording is an excellent technique to characterize pore insertion events and can potentially provide a comprehensive characterization of the pore-like properties of oligomeric αS. A disadvantage of the planar bilayer technique is that it is extremely sensitive and one has to ensure the data is reproducible. Although we have sometimes observed pore-like events, these events were not easily reproduced. The instability of planar bilayers composed of negatively charged lipids, even in the absence of oligomers, posed major problems. We have not seen evidence for an increased permeability without channel events [10], which is thought to be caused by membrane thinning or a change of the membrane dielectric structure upon oligomer adsorption [26]. However recent evidence indicates that the increased permeability levels reported in the literature may be caused by the presence of residual amounts of solvents that are used in some protocols of oligomer preparation [27]. The results from the planar lipid bilayer technique thus seem inconclusive. Experimental conditions that optimize the membrane stability and improve the protein insertion need to be identified.

To overcome these difficulties and to identify mechanistic aspects of the permeabilization process, several assays based on LUV and GUV systems were used. Confocal microscopy on GUVs shows that the disruption process is fast and without changes to vesicle morphology. Results from DLS experiments confirm that LUVs stay largely intact upon oligomer interaction. Additionally, LUVs containing the dye quencher pair HPTS/DPX were used to show that vesicle disruption occurs through an all-or-none mechanism. These data led us to hypothesize that vesicle permeabilization might be a transient event. In such a process defects occur upon protein adsorption, but are healed when the membrane reaches a new equilibrium state. In order to measure the permeabilization, an assay was developed based on the quencher DPX and a derivative of the dye HPTS conjugated to high molecular weight dextrans. This assay allows measuring the membrane permeability at different equilibration times after addition of the protein. In the absence of calcium however membrane defects were too large, and even the 70 kDa dextran was able to escape the LUV interior. It is unlikely that the inner radius of the hypothesized αS pore is large enough to allow diffusion of the 70 kDa dextran [16], [17], [18], which is reported to have a 58 Å Stokes radius [28]. Thus in the absence of calcium, disruption of POPG vesicles most likely does not occur through a pore-like mechanism but rather through large defects.

These defects could be caused by the instability of the negatively charged LUVs to protein adsorption. Many different unrelated proteins were found to cause calcein efflux from POPG LUVs if added in sufficient amounts. It has indeed been reported that vesicles containing high amounts of negatively charged vesicles are ruptured more easily by mechanical forces [29]. However, the disruption of the vesicles is not merely related to the vesicle instability but also to the structure of oligomeric αS. Compared to the different proteins added to the vesicles, disruption by oligomeric αS occurred in a concentration range more similar to melittin, which is a well known membrane disruptive protein. The finding that other proteins were less efficient in permeabilizing the membrane could be due to a difference in binding affinity for the POPG LUVs. However, the mode of binding is clearly important. From our experiments we have observed that monomeric αS has a higher lipid affinity but does not disrupt the vesicles as efficiently. Under specific conditions monomeric αS can even stabilize the membrane [30]. Therefore, the oligomers should contain structural properties that make it highly destabilizing to the bilayer integrity.

We have previously chosen to characterize the process of membrane permeabilization by oligomeric αS in the absence of calcium to reduce the complexity of the system [14], [15]. Both αS as well as negatively charged membranes are affected by calcium ions [31], [32]. At low concentrations, calcium ions already exhibit a pronounced impact on lipid packing and induce lipid ordering especially in negatively charged bilayers. We have previously shown that lipid ordering makes lipid vesicles more stable against membrane disruption by αS oligomers [15]. However, the selectivity of solute efflux on which the αS pore hypothesis is primarily based, was measured in the presence of calcium [16]. The findings presented in this article are in good agreement with this previous report. In the presence of calcium a size selectivity for marker diffusion across the POPG membrane was observed. The addition of oligomeric αS to LUVs did not induce the release of 3 kDa dextran-PTS. In contrast, the quencher DPX (422 Da) was able to cross the membrane and quench the encapsulated fluorescent dextran conjugate. However, care should be taken in considering these findings as direct evidence for the pore model. Non-equilibrium processes still contribute to the disruption process, which points to the presence of membrane defects. Membrane permeability is largest immediately after the addition of oligomeric αS. Subsequently, the permeability of the membrane goes down to a plateau level after equilibration. For a pore-like mechanism one would expect that the initial permeability is lowest and subsequently goes up to a plateau level, since the protein first has to bind and insert into the bilayer. An alternative explanation to the pore model is that in the presence of calcium, leakage still occurs through defects due to bilayer instability. The average size and lifetime of the defects might be related to the degree of membrane instability and membrane stress. In fact, even protein free lipid bilayers are known to contain unstable defects with finite sizes under conditions of stress [33]. Thus, such a mechanism might be possible and would also explain our observations of single channel like events with inconsistent conductance levels.

Whether these findings are valid for oligomers prepared by different methods is not clear. Recently, a number of methods that result in membrane active αS oligomeric species have been reported. Danzer et al. have used Fe^3+^ to induce αS oligomers that were toxic and showed pore-like single channel recordings [8], [24]. Another recent study used cold denaturation of αS fibrils to prepare toxic oligomeric αS species, which also showed pore-like characteristics [23]. An alternative route to pore formation might be the aggregation of monomeric αS on the membrane [34]. Membrane bound monomeric αS could be more aggregation prone [35] and thus form pores on the membrane [17], [36]. Establishing if these different results are caused by structurally distinct oligomeric species is an important future challenge. The differences in membrane interactions would point to possible different oligomer conformers. For instance we have never observed any interaction of the oligomeric species with zwitterionic lipids [14], [15], whereas others have reported pore formation of αS oligomers in membranes composed of such lipids [17], [23], [36]. One of the problems in finding answers to these questions is that a structural characterization of the oligomeric species is challenging. We have recently used tryptophan fluorescence to provide insight into the structural organization of αS oligomers [25]. However, characterizing the structure in molecular detail is limited by the low concentration at which these species occur and possibly the structural heterogeneity of the oligomeric species. In addition, as we show in this paper, it is crucial to fully characterize the membrane permeabilization of the oligomers using a number of different techniques in order to elucidate details at the molecular scale.

In summary, the αS oligomers prepared according to our protocol are a membrane active species, and have a destabilizing effect on the bilayer integrity. The produced αS oligomers are relatively efficient at disrupting the vesicles compared to other proteins, which is most likely linked to structural features of the oligomeric complex. Although we cannot exclude that αS forms membrane pores, lipid bilayer permeabilization by αS oligomers appears to be dominated by transient non-equilibrium processes. Membrane defects appear due to protein adsorption and intrinsic vesicle instability. Therefore, extreme care should be used when using highly negatively charged vesicles as a model system for detecting oligomer membrane permeabilization.

## Materials and Methods

### Purification of monomeric and oligomeric αS

Purification of αS and preparation of oligomeric species was performed as previously published [15]

### Planar lipid bilayer measurements

Solvent free planar lipid membranes were formed by the joining of lipid monolayers according to the method of Montal and Mueller [20]. A small aperture (40–70 µm) was punched through a 40 µm thick Teflon film by an electric discharge. The film was clamped between two Teflon cuvettes and the aperture was pretreated on both sides with 3 µl 1% (v/v) hexadecane in hexane. After evaporation of the hexane, 250 µl of 10 mM HEPES pH 7.4, 150 mM KCl was added to both sides of the film. Lipid monolayers were formed on top of the solution by adding 1 µl of lipids in pentane (5 mg/ml). The solvent was allowed to evaporate for 10 min. Lipid bilayers spontaneously formed by lowering and raising the liquid level on one side of the aperture. Membrane conductance was measured by two Ag/AgCl electrodes on the *cis-* and *trans-*side of the membrane. The electrodes were connected to an Axopatch 200B amplifier (Axon instruments). Signals were digitized using a Digidata 1440A (Axon instruments) digitizer and stored on a personal computer for further analysis. The cuvette and amplifier head stage were located in a Faraday cage inside an acoustically isolated room to minimize environmental noise contributions. Membrane quality was assessed by measuring bilayer capacitance and conductance. The data was analyzed using the QUB software package [21].

### Preparation of GUVs

Around 0.05% DOPE-Rhod was added to the membrane lipid composition. Approximately 1 mg of total lipid in chloroform was deposited in a glass vial and dried using nitrogen gas. Residual chloroform was removed by drying under vacuum for 4 hours. GUVs were subsequently created by gentle hydration. The lipid film was hydrated for 6 hours at room temperature in 500 µl of sucrose solution containing 20 µM of HPTS of equal osmolarity to 10 mM HEPES pH 7.4, 150 mM NaCl.

### Confocal Microscopy

Confocal microscopy was performed on a Zeiss LSM 510 confocal microscope. DOPE-Rhod was excited at 543 nm using a green HeNe laser and HPTS was excited using the 488 nm Argon laser line. Emission from both dyes was measured simultaneous using the appropriate dichroic mirrors and filter sets. HPTS filled GUVs were diluted to approximately 30 µM lipid concentration in 10 mM HEPES, pH 7.4, 150 mM NaCl and 10 mM DPX. A solution of oligomeric 5µM αS in 10 mM HEPES pH7.4 and 150 mM NaCl was injected at one end of the imaging chamber and allowed to slowly diffuse though the chamber (final concentration ∼1.5 µM). GUVs were followed by confocal microscopy in continuous imaging mode. Imaging conditions were optimized with respect to the temporal resolution.

### Preparation of LUVs

1-Palmitoyl,2-oleoyl phosphatidylglycerol (POPG) was obtained from Avanti Polar lipids and used without further purification. Large unilamellar vesicles (LUVs) were prepared by extrusion. First a thin lipid film was formed by drying around 0.5 mg of lipid in a glass tube using a gentle stream of N2. Trace amounts of solvent were removed by drying under vacuum for at least 4 hours. The lipid film was then hydrated by adding a solution, 150 mM NaCl, 10mM HEPES, pH 7.4 containing the solute to be encapsulated in the vesicle, unless noted otherwise. Hydration was continued for 1 hour, with vortexing approximately every 15 minutes. The sample was subsequently subjected to 5 freeze-thaw cycles by dipping into liquid nitrogen and thawing above the lipid phase transition temperature. The resulting solution was extruded 11 times through a polycarbonate membrane filter with a 100 nm pore size. This procedure was repeated once with a new filter. Unencapsulated dye was separated from the vesicles by gel filtration through a PD10 column packed with Sephadex G-100 (GE) unless noted otherwise. The total phospholipid concentration was determined according to the protocol of Chen *et al.* [37].

### Calcein Efflux assay

The calcein efflux assay was performed as previously published [15]

### DLS

The DLS experiments were performed on a Malvern Zetasizer Nano zs. Oligomeric αS and POPG LUVs in 10 mM Hepes pH 7.4 and 150 mM NaCl were mixed to a final concentration of 20 µM lipid and 1 µM αS oligomers and placed in a 3mm path length quartz cuvette. Light scattering was measured over 15 minutes in 5 second runs. Data analysis was performed using the Malvern DTS software.

### Fluorescence re-quenching assay

The assay was performed similar to the protocol as published by Ladokhin *et al.* [22], with the modification that the dye 8-Hydroxypyrene-1,3,6-trisulfonic acid (HPTS) was used in combination with the quencher p-Xylene-bis(N-pyridinium bromide) (DPX). Both dyes were obtained from Sigma-Aldrich. Quenching of HTPS fluorescence by DPX occurs via a static quenching mechanism, that is, the fluorescence intensity is reduced but not the fluorescence lifetime. Fitting a weak association model to the intensity data results in an association constant *K*_a_∼2 mM^−1^. The assay tries to discern two different scenarios: If 50% content release from vesicles is observed is this caused by half of the vesicles releasing all their content (all-or-none mechanism) or by all vesicles releasing half of their content (graded release). Considering the concentration range probed, the quenching ratio of HPTS is dependent on the DPX concentration. If vesicles are disrupted by an all-or-none mechanism, the quenching ratio inside the vesicles that are still intact has not changed. For graded release the quenching ratio inside the vesicles changes due to the efflux of DPX (assuming that DPX and HPTS have a similar probability to be released from the vesicle). Thus by estimating the quenching ratio of the vesicle interior (*Q_in_*) as a function of vesicle disruption represented by the fraction of released dye *f_out,_*, it is possible to discern graded (*Q_in_* changes with *f_out_*) from all-or-none release (*Q_in_* remains constant with respect to *f_out_*). Both *Q_in_* and *f_out_* are experimentally accessible through the following equation:
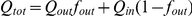
In a typical experiment the protein is allowed to interact with vesicles causes a fraction of dye efflux or *f_out_* and a certain value of *Q_in_*. After leakage from the vesicles has reached a plateau value, DPX is added in aliquots, which leads to several values of *Q_out_* that are determined from a calibration curve. From measuring the fluorescence intensity after each addition of DPX *Q_tot_* can be calculated, since *Q_tot_ = F_max_/F*. The maximum possible value of fluorescence (*F_max_*) is determined by breaking all vesicles through the addition of triton X-100 after the final DPX addition. By plotting Q_tot_ as a function of Q_out_, the values for *f_out_* and *Q_in_* are determined from the slope and intercept of a linear fit.

POPG LUVs filled with 1mM HPTS, 2.5 mM DPX in 10 mM HEPES, pH 7.4 and 150 mM NaCl were prepared by extrusion. HPTS fluorescence was measured on a Varian Eclipse fluorimeter, with the excitation wavelength at 440 nm (10 nm slit) and emission was measured from 480 nm to 530 nm (10 nm slit). Vesicles were mixed with αS oligomers at several protein concentrations and a final lipid concentration of 25 µM. After 30 minutes the HPTS fluorescence was measured and subsequently DPX was added in aliquots to a final concentration of 3mM. After each addition the HPTS emission spectrum was measured. After the final DPX addition 0.5% (weight/volume) Triton X-100 was added and a spectrum was measured.

### Transient permeabilization assay

For the transient permeabilization assay, amine modified dextran molecules were labeled with an isothiocyanate derivative of the dye HPTS. The resulting dextran-PTS conjugate could be effectively quenched with DPX. Dextran (3 kDa, 10 kDa and 70 kDa) functionalized with amino groups was obtained from Invitrogen. The dye 8-Isothiocyanatopyrene-1,3,6-trisulfonic acid trisodium salt was obtained from Sigma-Aldrich. For dextran labeling around 2.5 mg of dye and 7.5 mg dextran were dissolved in 400 µl sodium bicarbonate buffer pH 9. After a two hour incubation, free dye was separated from the dextran bound dye by size exclusion chromatography. For the 3kDa and 10 kDa dextran a Superose 12 column (GE) was used. The 70 kDa dextran was separated on a Superdex 200 column (GE), with 10 mM Hepes pH 7.4 and 50 mM NaCl as elution buffer. The fractions containing dye labeled dextran were pooled and concentrated using a Vivaspin (Sartorius) centrifugal concentrator with the appropriate molecular weight cutoff. For hydration around 150 µl of the resulting labeled dextran solution was added to 2 mg of dried POPG lipids. After extrusion the LUVs were separated from the free dextran by size exclusion chromatography on a Superdex 200 column. Fluorescence from the dextran-PTS filled LUVs was measured on a Varian Eclipse fluorimeter. The excitation wavelength was 430 nm with a 5 nm slit width and emission was measured at 500 nm with a 20 nm slit width. In a typical experiment the dextran-PTS emission was followed over time at t = 0, 50 µl of LUVs were added to 50 µl protein solution, to a final concentration of 250 µM phospholipids and 1.5 µM αS oligomers. After varying reaction times 5 µl of 100 mM DPX was added.

## Supporting Information

Movie S1 Confocal microscopy movie of POPG GUVs filled with the dye HPTS (green) in a flow chamber. The quencher DPX was present on the outside of the vesicles. The GUV membrane was stained with DOPE-Rhodamine (red). An aliquot of oligomers was introduced into the chamber (top of the image frames), and imaging was continued.
(67.64 MB AVI)
